# Experimental Investigation of Engine Valve Train Friction Considering Effects of Operating Conditions and WPC Surface Treatment

**DOI:** 10.3390/ma16093431

**Published:** 2023-04-28

**Authors:** Muhammad Usman Bhutta, Muhammad Huzaifa Najeeb, Muhammad Usman Abdullah, Samiur Rahman Shah, Muhammad Khurram, Riaz Ahmad Mufti, Kiyotaka Ogawa, Jawad Aslam, Rehan Zahid, Mian Ashfaq Ali, Muazzam Arshad

**Affiliations:** 1 School of Mechanical & Manufacturing Engineering (SMME), National University of Sciences & Technology (NUST), Campus H-12, Islamabad 44000, Pakistan; 2 Department of Mechanical Engineering, National University of Technology (NUTECH), Main IJP Road, Sector I-12, Islamabad 44000, Pakistan; 3 Fuji Manufacturing Co., Ltd., 5-2-24 Matsue Edogawa-ku, Tokyo 132-0025, Japan; 4 Chemical Engineering Department, Faculty of Chemical, Mechanical and Industrial Engineering, University of Engineering & Technology, Peshawar 44000, Pakistan

**Keywords:** engine valve train, cam–roller interface, friction, WPC, camshaft speed, operating temperature

## Abstract

Reduction in friction ensures fuel economy, control on emissions and durability of components in internal combustion engines. A modern gasoline internal combustion engine was instrumented to determine the friction values at the cam–roller interface considering the effects of surface treatment and engine operating state. A series of tests under different operating speeds and lubricant inlet temperatures were undertaken using both an original surface roller and a Wonder Process Craft (WPC) surface-treated engine roller. The results clearly revealed a substantial reduction in friction magnitude for the WPC surface-treated engine roller in comparison to the original roller while operating under similar conditions, indicating their strong potential for employment in engines. An increase in friction with the rise in temperature was also observed for both types of rollers, whereas increased lubricant entraining velocity due to higher operating speed had the opposite impact. A considerable reduction in frictional drive torque ranging from 8% to 28% was observed by employing the WPC-treated roller in comparison to original/untreated roller at various operating conditions, which signifies the strong potential for employment of WPC surface treatment in the roller/follower valve train engines.

## 1. Introduction

There is constant pressure on the automotive industry to improve the fuel economy and to control the emissions of internal combustion engines while ensuring reliability of tribological components in engines. Reduction in friction is the best way to achieve these goals. Advanced lubricant formulations and employment of engineered surfaces are being pursued relentlessly in the automotive sector to reduce the engine friction and wear. The cam–follower pair in an engine valve train is often exposed to harsh operating conditions in the form of starved lubricity, high contact loadings, raised operating temperature and higher sliding velocities, which can cause excessive friction and can significantly affect the durability of components. Of the total frictional loss in an internal combustion engine, 6% to 35% is attributed to the valve train [1,2]. At lower engine speeds and higher operating temperatures, the valve train’s frictional losses become of relatively greater importance [1,3]. Due to wear and consequently reliability problems, the engine valve train has proven to be the most difficult to design and lubricate effectively [4]. Presently, there is an increased trend in using low-viscosity oils in engines to reduce sliding friction and to achieve enhanced fuel economy. The same has pushed the lubrication mode at the cam–follower interface further towards the mixed and boundary lubrication regimes, thereby raising concerns about the durability of the mating engine components.

Direct-acting bucket tappet and end-pivoted roller follower configurations are extensively used in modern engines due to their superior performance. Significant research has reported in the past on the frictional and wear characteristics of bucket tappets valve trains [5,6,7,8,9,10,11,12]. However, little to no work has been performed/reported on the friction measurement at cam–roller contact, particularly in-end pivoted roller finger follower valve trains due to involvement of experimental difficulties. In the past, it has been reported [13] that the valve train friction can be reduced by about 50% by employing a roller follower. It was identified [14] that frictional losses were reduced by 80% using a roller follower as compared to a flat-faced follower. The research of [15,16] analyzed the friction of a Cummins L-10 engine valve train with a roller follower and identified that the frictional loss was mainly due to the mixed lubrication regime. In a creep study [17] at the cam–roller interface reported that the contact experiences sliding and rolling friction, where the rolling friction dominates. It was also reported that [18,19] the sliding of rollers on the camshaft surface can increase the friction in a roller follower valve train.

The majority of the experimental research work on friction measurement in roller follower valve trains reported in the past has been performed on highly modified test rigs, which may restrict the capture of the true picture of the phenomenon under investigation. A recent shift in the engineering oriented towards surface texturing, surface coatings, surface treatments and nanoparticles has shown significant improvement in durability, friction, wear and overall performance of mating machine parts [20,21,22,23,24,25,26,27,28,29]. Real production engines have been used for investigating the tribological behavior of various engine components, parts and assemblies [8,19,30,31]. Moreover, employment of engineered surfaces is also being pursued vigorously in the automotive sector to reduce friction and improve efficiency, life and durability of components. The only work that has been reported on the analyzing the effect of WPC treatment in a production engine is [32], which showed the benefits of this treatment in reducing roller slip on a real production engine tested under motored conditions. The results of [32] clearly present the advantages of using this surface treatment method, and WPC requires further experimental investigation. Thus, an elaborated research work to measure friction at the cam–roller interface considering sensitivity to surface treatment under actual operating conditions becomes essential.

**Figure 1:**
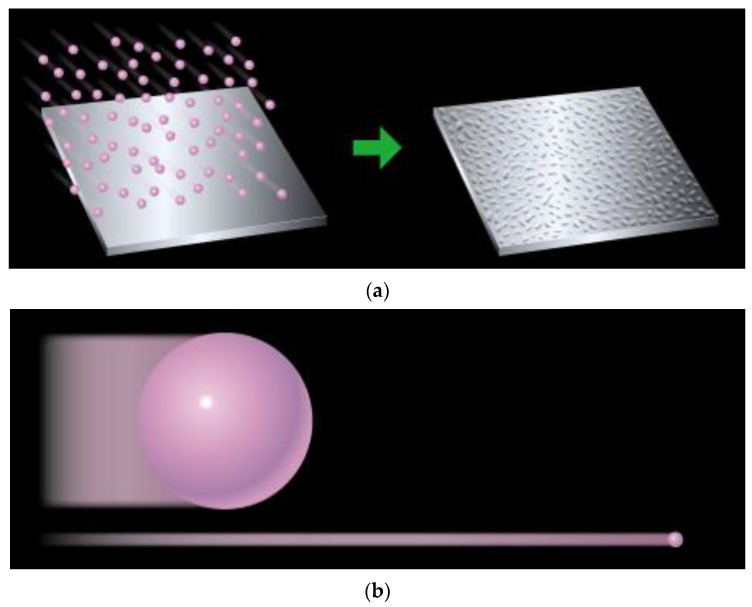
(**a**) WPC process, (**b**) Shot peening (**left**) & WPC (**right**). Reprinted/adapted with permission from [36]. 2023, FUJI KIHAN CO.,LTD [37].

The WPC (Wonder Process Craft) surface treatment method has been used for a number of years in Japan. Limited details are available about the WPC process, which has over 40 patents [33,34]. The WPC surface treatment process is analogous to shot peening. As represented in Figure 1a, WPC treatment is a technology that involves a jet of particles mixed into a compressed gas, which is bombarded onto a metallic surface at high speed [35]. Fuji Kihan Co., Ltd., Tokyo, Japan, developed a surface treatment procedure in which the effects of forging and heat treatment were attained by injecting particles within a duration of tens of seconds to a time span of several minutes at high speeds, having a diameter of 50 microns, 1/3000 size in volume in comparison to those used in conventional shot peening methods, which involve the use of particles of around 600–800 mm in diameter [33,34,35]; a comparison between the particle size has been shown in Figure 1b.

This surface treatment method emulates a strengthening process achieved during forging, heat treatment, penetrant diffusion and plating by instantaneous repeated rapid heating and cooling. The repetitive rapid heating and cooling is achieved by injecting the fine particles onto the metallic surface [37]. The metallic structure becomes extremely durable through densification, miniaturization and the alloying process [37].

Intrinsically engraved machining fine grooves on metallic surfaces are transformed into micro-dimples by the impact of ultrafine media through WPC treatment, which then act as reservoirs for oil [32,34]. The transformed and thoroughly compacted surface overcomes the issues of brittleness that is normally faced when metallic parts are hardened [34,35,37]. WPC treatment helps reduce sliding friction when applied on pistons, cam shaft and crank shafts [36,38]. Friction reduction in WPC-treated mating surfaces is obtained by creating a unique micro-dimple pattern on the surface [32,37]. WPC corrects pinholes and blowholes and in casted products, increases resistance to wear, chipping resistance and pitching resistance [39].

**Figure 2:**
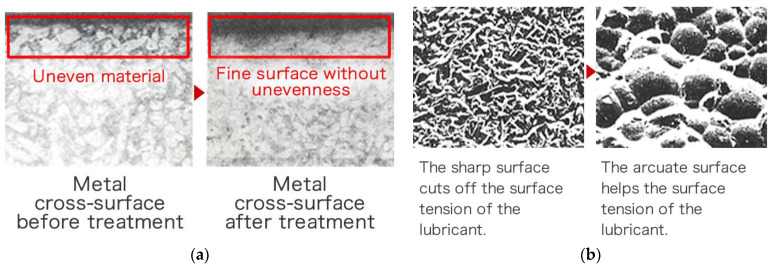
(**a**) Thermal treatment effect; (**b**) micro-dimple effect. Reprinted/adapted with permission from [36]. 2023, FUJI KIHAN Co., Ltd. [40].

To summarize, WPC surface treatment technology has effects of enhanced impact resistance, better surface life, lower slide resistance, increases the power and provides better fuel efficiency, and shortens the initial engine running-in period [33,34,35,36,38,39]. WPC enhances surface hardness, provides better adhesion of films and results in low-temperature brittleness prevention and corrosion prevention [33,34,35,36,38,39]. Images of WPC-treated metallic parts are shown in Figure 2.

In this research, Wonder Process Craft (WPC) surface treatment was applied to an end-pivoted roller finger follower and an internal combustion engine was instrumented to determine the friction at cam–roller contact in a Toyota Prius 1NZ engine head. A comprehensive test program at different camshaft operating speeds and various oil inlet temperatures was undertaken. Both original and WPC surface-treated rollers were used to determine the values of friction at cam–roller contact, thereby providing opportunity to analyze the impact of the surface treatment on friction reduction. No modification of components was ensured to obtain the true picture of the phenomenon under investigation. It is strongly believed that the reported research work will be very valuable to enhancing the tribological efficiency of roller follower valve trains. This paper aims to highlight an effective way, based on surface treatment of tribological components, of reducing the friction in engine valve trains.

## 2. Experimental Setup

### 2.1. Engine

The experiments were performed on the cylinder head of a Toyota Prius 1NZK gasoline engine. It is a 4-cylinder, 1.5 L, 16-valve engine with a variable valve timing-intelligent (VVT-i) system. Each cylinder consists of two intake valves and two exhaust valves, which are directly operated by twin camshafts.

The angle of these valves is set at 33.5°; therefore, the cylinder head is quite compact. The valves are operated by an end-pivoted roller finger follower with hydraulic lash adjusters. Both the intake and exhaust camshafts are driven through a timing chain. An oil passage is provided inside the camshafts so that oil can be supplied to the intelligent (VVT-i) system. The camshaft and cam roller interface are sprayed with oil through a spray channel located in the cover of the engine head for lubrication purposes.

### 2.2. Test Bench

**Figure 3:**
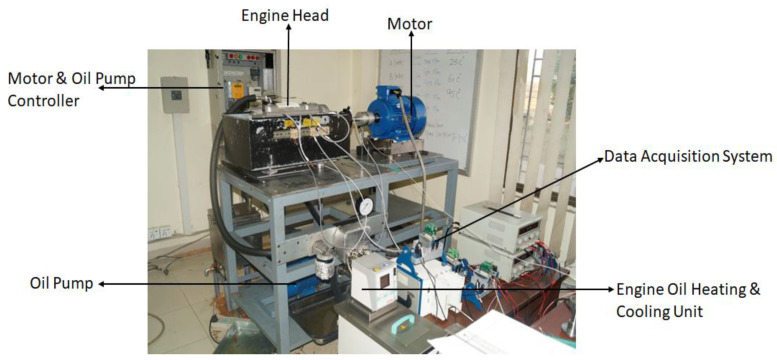
Engine test bench.

**Figure 4:**
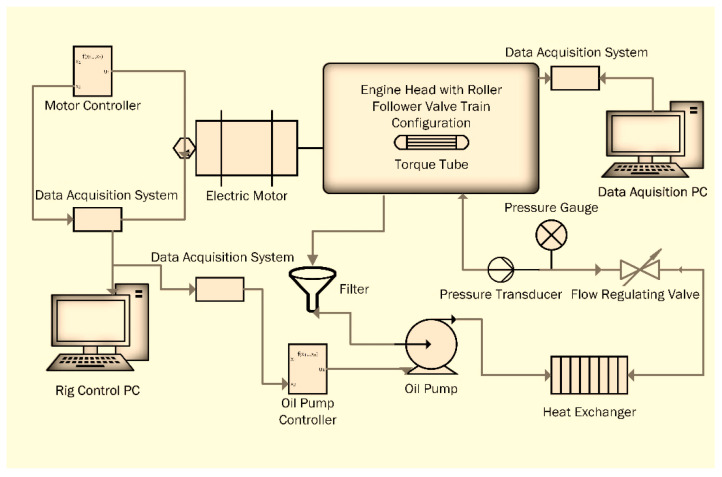
Schematic diagram of engine test bench.

A flexible engine test bench (Figure 3) was developed for the tests, and the schematic of the test bench is shown in Figure 4. It comprised two electric motors, an oil pump, oil heating/cooling system, motor controllers and an optical encoder. The cylinder head was bolted securely onto the test plate, which was then attached to a rig stand in order to minimize vibrations. The camshaft was driven with a 5.5 kW, 3-phase induction motor. The camshaft was connected to the motor with the help of an axial plug-in type steel bellows coupling. The purpose of using this specific coupling was to transmit the torque without any backlash and to provide compensation for radial, angular and axial misalignments. The speed of the camshaft could be varied up to 3000 rpm and was controlled through a microprocessor-based programmable AC motor controller. The controller was set up in a closed-loop vector mode feedback mechanism through an encoder so that 100% standstill torque could be provided. A high resolution 760 series pull-up type British optical encoder that provided 720 incremental pulses per revolution was mounted at end of the electric motor. The encoder was used in the determination of the cam–crank angle and acted as an external clock for the data acquisition system so that the instantaneous friction torque reading could be synchronized with the camshaft angle. The top of cam lift was determined using a dial indicator with an accuracy of ±0.4°. The index pulse of the optical encoder was synchronized to appear at the cam’s nose. The incremental pulses of the encoder were used as an external trigger to acquire the signal from the strain gauges at every 0.5°, providing 720 readings in one complete rotation. The index pulse was read separately as an analog input. For oil circulation through the engine, a 180-bar oil pump was used. The pump was driven by a 1.5 kW electric motor. Temperature of the oil was controlled with the help of an in-line plate type heat exchanger, which was connected to an external heating and refrigerant circulation unit. A Piezo-resistive pressure sensor was used to measure the oil pressure, whereas a k-type thermocouple was attached at the oil inlet of the engine head gallery for measuring the oil temperature. In order to prevent heat loss, all of the oil-circulating pipes were properly insulated.

The oil pressure was also controlled through a separate microprocessor-based programmable controller. Feedback proportional integral derivative (PID) controls were used to control the engine parameters, i.e., the camshaft speed, the lubricant pressure and the oil temperature.

## 3. Instrumentation and Calibration

### 3.1. Torque Tube

**Figure 5:**
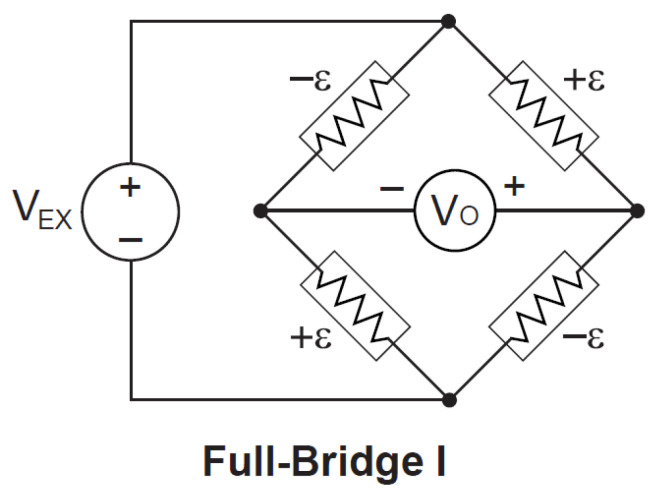
Schematic of full bridge strain gauge circuit.

**Figure 6:**
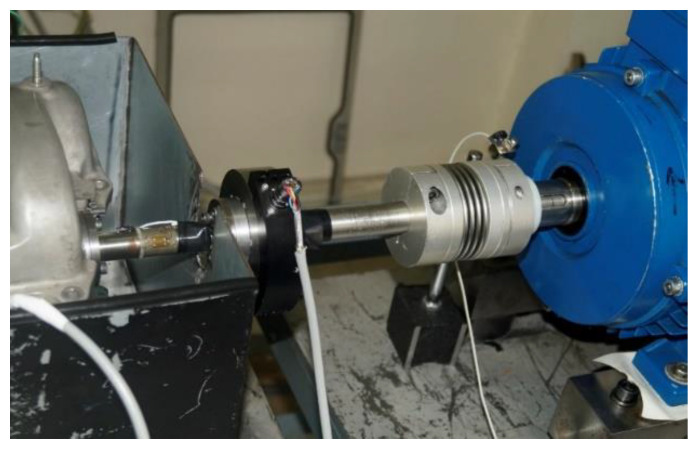
Attachment of torque tube with motor.

In this research project, a specially designed torque tube was used to measure the valve train’s friction. The torque tube was conceived in a way that it could be coupled with camshaft without any modification. For this purpose, special flange adopters were designed and manufactured. With the help of these adopters, the torque tube was attached to the exhaust camshaft. High-resistance, two-element 90° torque gauges were positioned on the torque tube at opposite sides so that the torsion and consequently the drive torque could be evaluated. The strain gauges were attached in a full bridge circuit configuration (Figure 5) so that the effect of temperature/thermal drift on the output electrical signal could be reduced. Michigan Scientific B6-2 slip ring assembly was used to handle strain gauge bridge and maintain proper drive arrangements, as shown in Figure 6.

### 3.2. Calibration Procedure

**Figure 7:**
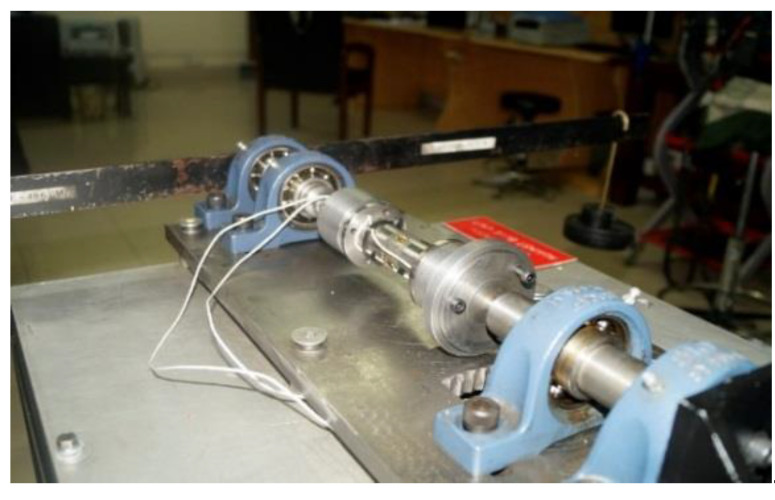
Torque transducer calibration bench.

Before using the torque tube for experimental tests, it was calibrated by applying torque using a calibration bench. The torque tube was fixed firmly between two shafts with flange adapters. These shafts were then fixed with self-aligning frictionless ball bearings, which in-turn were attached to the work bench. One end of the torque tube was made stationary/static by bolting the attached shaft to a fixed support. A bar along with a weight-hanging arrangement was attached to the other end (Figure 7).

**Figure 8:**
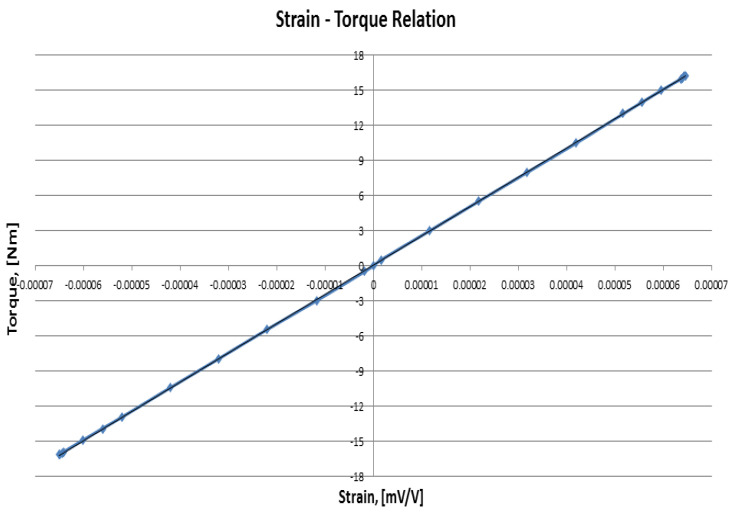
Torque transducer calibration curve.

The calibration of the torque tube was carried out by taking 12 readings in both clockwise and counterclockwise directions over a span of ±20 Nm. A relationship was developed between the applied torque and the output strain values by measuring the output strain against each value of the applied torque. For each step, an average of three calibration readings were taken and a relation was developed as shown in Figure 8.

The slope of this straight line was calculated, which provided the equation relating the output strain with the applied torque. The frictional torque values of the ball bearings were taken as negligible compared to the applied torque.

## 4. Materials and Methods

**Figure 9:**
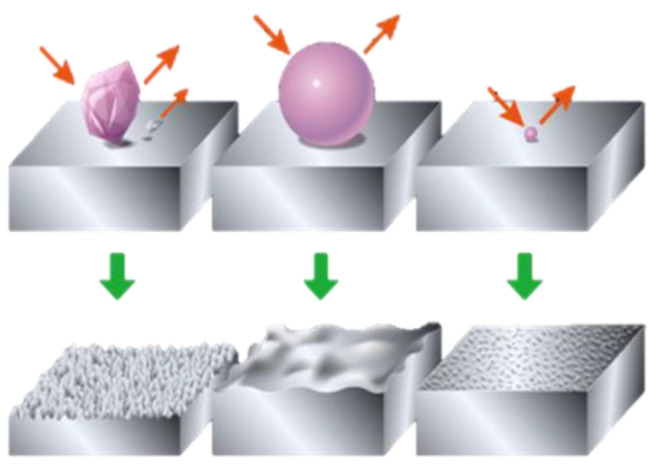
WPC treatment process. Reprinted/adapted with permission from [36]. 2023, FUJI KIHAN Co., Ltd. [38].

To investigate the effects of surface treatment of friction, WPC treatment was carried out on the roller race of a Toyota Prius 1NZK engine valve train, which is closely related to the shot peening process [35]. In WPC treatment, spherical ceramic media of nearly 50 microns size were bombarded on a metal surface at near-sonic speed, which formed a hard micro-dimpled surface, as shown in Figure 9.

**Figure 10:**
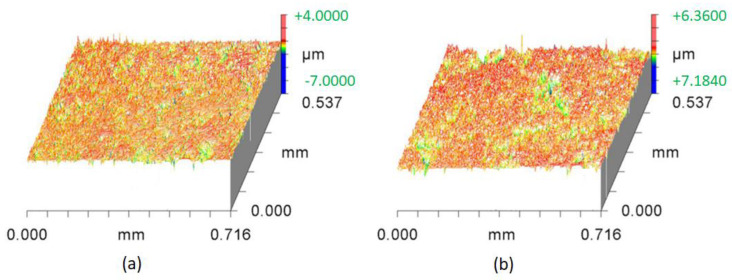
Three-dimensional surface optical profilometry plots: (**a**) original untreated roller; (**b**) WPC-treated roller.

**Figure 11:**
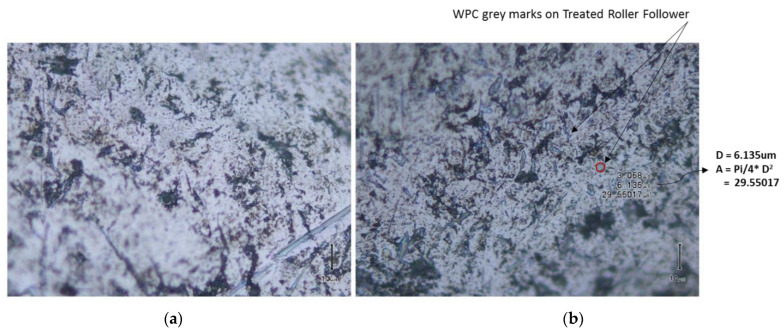
(**a**) Original untreated roller; (**b**) WPC-treated roller.

This bombardment produces surface compressive stress, plastic deformation and grain refinement [36]. As a result, the fatigue strength, friction and stress corrosion and fracture resistance of the component is improved, considerably. Due to the small mass of the media, the affected area of WPC treatment is less than 0.0254 mm [35,40]. Due to WPC treatment, the surface roughness increases, which consequently results in increased wettability [41]. According to the Wenzel relation [42], the ability of liquids to retain their contact with a solid surface is enhanced by increasing the values of surface roughness up to a certain limit. A 3D optical interferometer was used to create three-dimensional surface profiles of both the treated and untreated roller as shown in Figure 10. The average surface roughness (Ra) of the untreated roller was found to be 0.204 µm, whereas the Ra of the treated roller was determined as 0.402 µm. Average surface roughness (Ra) of mating metallic parts is known to have an influence on overall friction and wear performance [43]. The average surface roughness (Ra) of the cam lobe was 0.160 µm. Scanning electron microscopy images of WPC-treated rollers are shown in Figure 11. The grey marks on the surface of the rollers clearly describe the evidence of the micro-shot peening spots.

## 5. Data Acquisition

An advanced high-speed data acquisition system (DAQ) was developed to measure the valve train’s friction. This DAQ system was based on National Instruments high-tech DAQ hardware and LabVIEW software. The entire data acquisition system was divided into two main groups. First group comprised different compact DAQ modules, which were used to control and monitor oil temperature, engine camshaft speed and oil pressure. The second group comprised an SCXI-1000 chassis with some SCXI modules whose purpose was to acquire and read the index and incremental pulses from the encoder along with the strain gauge signal. The incremental encoder pulses were used as an external trigger to acquire the signal. The encoder provided an incremental pulse after every 0.5 degrees of the cam angle; therefore, the circuitry was designed in such a way that the strain value from the torque tube could recorded after every 1 degree of the cam angle. High-quality slip ring assembly was used to pass output strain signals from the rotating torque tube to the SCXI terminal block. The strain gauges were used in a full bridge configuration with an excitation voltage of 5 Volts. Simultaneous sampling was used to acquire all the data. The process of controlling, monitoring and logging the complete data was performed using a custom-designed graphical user interface program in LabVIEW software.

## 6. Experimental Procedures

The experiments were performed for the exhaust-side camshaft only, as both inlet and exhaust cams are identical. The experiments were conducted in two phases. In the first phase, experiments were performed on the untreated roller, whereas in the second phase, a WPC-treated roller was used. For both phases, the friction was measured at varying lubricant temperatures of 25 °C, 60 °C and 95 °C and camshaft speeds of 600 rpm, 1200 rpm and 1800 rpm, respectively. A base oil of Group IV with 4cSt kinematic viscosity was used. The recent trend of thinner oil application to engines by OEMs made this base oil the most suitable one for this experimental study.

It has been previously reported by [1,2,3,5,9,10,44] that despite using temperature-compensated gauges, on elevated temperature, the signal levels of the transducers start to drift at zero torque. To counter this effect, a methodology was developed and reported by [10], which has been employed in this experimental testing as well. According to this methodology, the engine lubricant has to be heated up to the desired temperature using an external refrigeration and heating unit and circulated through the engine for 2 h, after which the built-in strain reading at idle condition is recorded and considered as the zero-torque value, which is then subtracted from the final results. The sampling rate was set using the incremental pulse from the encoder. The encoder provided an incremental pulse after every 0.5 degrees of the cam angle therefore the circuitry was designed in such a way that the strain value from the torque tube could be recorded after every 1 degree of the cam angle. Each data set was recorded between the interval of two index pulses of the encoder, which occurred after every 360°. After following these procedures, tests were performed, and the corresponding data was logged and processed.

**Figure 12:**
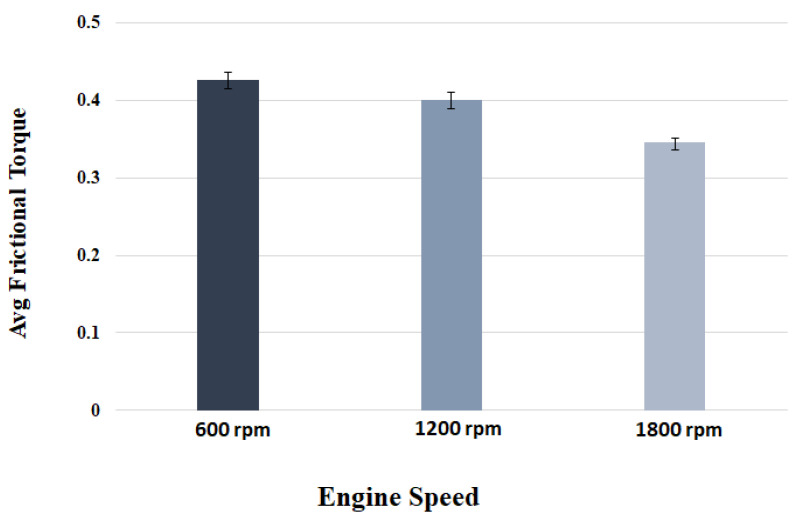
Repeatability test at 25 °C.

**Figure 13:**
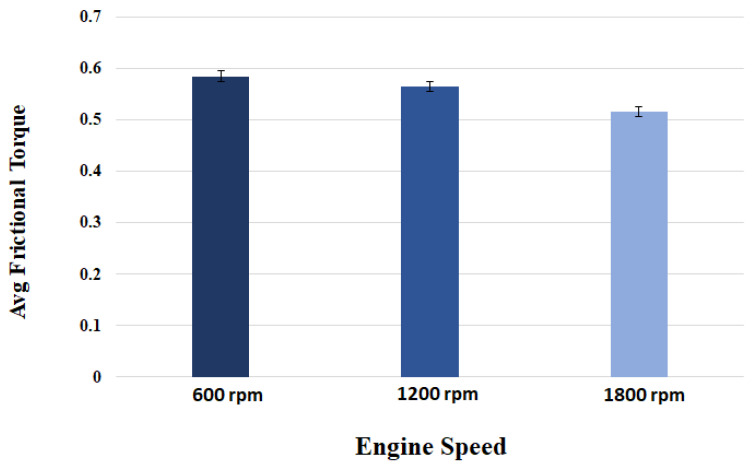
Repeatability test at 95 °C.

During the tests, engine operating conditions were continuously monitored. Repeatability was performed on the untreated rollers and the test results were found to be under 0.1% in variation, as shown in Figure 12 and Figure 13, indicating the greater suitability of a complete measurement system for the said research project.

## 7. Results and Discussion

The data acquired from the torque transducers depicted a characteristic waveform. Since all the tests were performed under motored conditions, the shape of the waveform remained constant, which exhibited high repeatability of the installed transducers. The instantaneous torque typically comprises frictional (which is a function of tribological performance) and geometrical torque components.

The geometrical torque emerges due to the valve’s opening and closing operations. In order to open the valve, force is applied against the resistance of the valve spring, and the energy generated from this process is consequently stored in the valve spring, which in turn is released to the camshaft during the closing of the valve. Therefore, the geometrical torque has equal positive and negative constituents. On the other hand, the frictional torque is significant during the valve’s opening but diminishes during valve’s closing. As a result, the waveform depicts more values on the positive side than the negative side. By taking the mean torque, the geometrical torque is effectively cancelled out, leaving behind only the frictional component.

**Figure 14:**
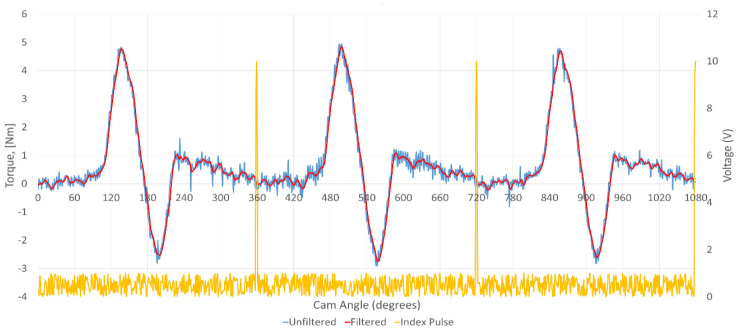
Result of applying Butterworth filter to the torque transducer result.

Since vibrations of low levels were present in the setup, some noise was therefore present in the test results, which was then removed by digitally filtering the data through a Butterworth filter. The cut-off frequency of the filter was set to 200 Hz and the order of the filter was set to two. Figure 14 shows the result of digital filtering the data obtained from the torque transducer.

**Figure 15:**
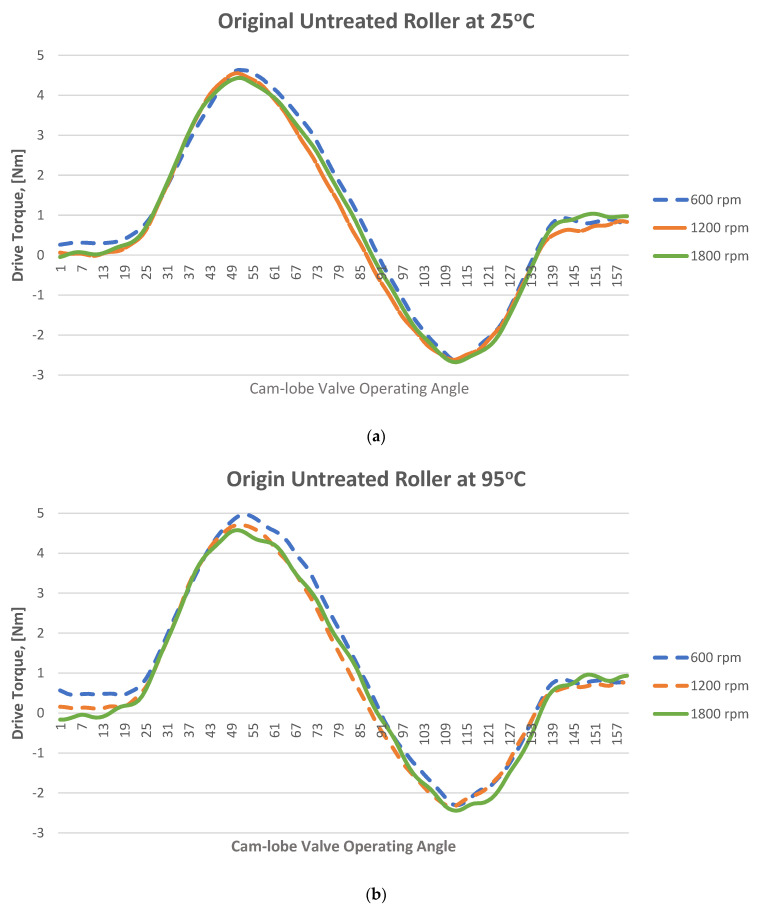
Experimental instantaneous drive torque at engine speed of 600 rpm, 1200 rpm and 1800 rpm at temperatures ff (**a**) 25 °C and (**b**) 95 °C; original roller.

**Figure 16:**
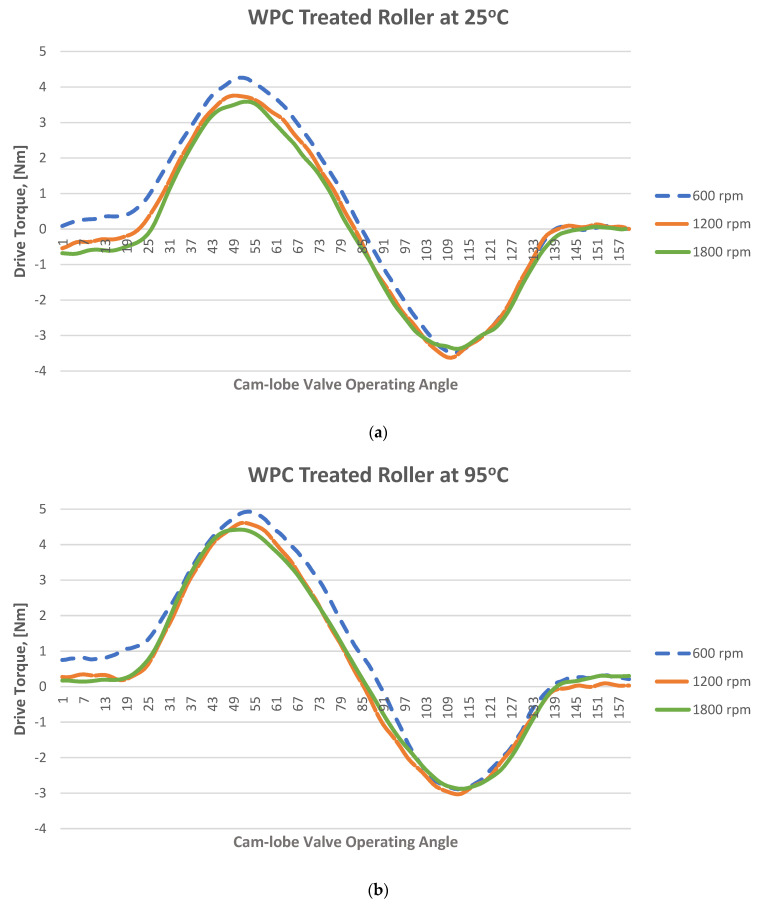
Experimental instantaneous drive torque at engine speed of 600 rpm, 1200 rpm and 1800 rpm at temperatures of (**a**) 25 °C and (**b**) 95 °C; WPC-treated roller.

The instantaneous friction profiles at the cam–roller contact of both the treated as well as untreated rollers at 25 °C and 95 °C are shown in Figure 15 and Figure 16, respectively. The geometrical torque emerges due to the valve’s opening and closing operations. To open the valve, force is applied against the resistance of the valve spring, and the energy generated from this process is consequently stored, which in turn is released to the camshaft during the valve’s closure. Therefore, the geometrical torque has equal positive and negative constituents. On the other hand, the frictional torque is significant during the valve’s opening but diminishes during valve’s closing. As a result, the waveform depicts more values on the positive side compared to the negative side. By taking the mean torque, the geometrical torque is ideally cancelled out, effectively leaving behind only the frictional component. Drive torque values corresponding to cam rise and fall corresponding to the cam’s nose area responsible for opening and closing of the valves were observed, and the data on the base circle of the cam lobe, which ranges from 0° to 90° and from 240° to 360°, did not significantly affect the drive torque values and this data was thus filtered out for all tests. Since vibrations of low levels were present in the setup, yielding some noise in the test results, they were removed by digitally filtering the data through a Butterworth filter. The cut-off frequency of the filter was set to 200 Hz and the order of the filter was set to 2.

**Figure 17:**
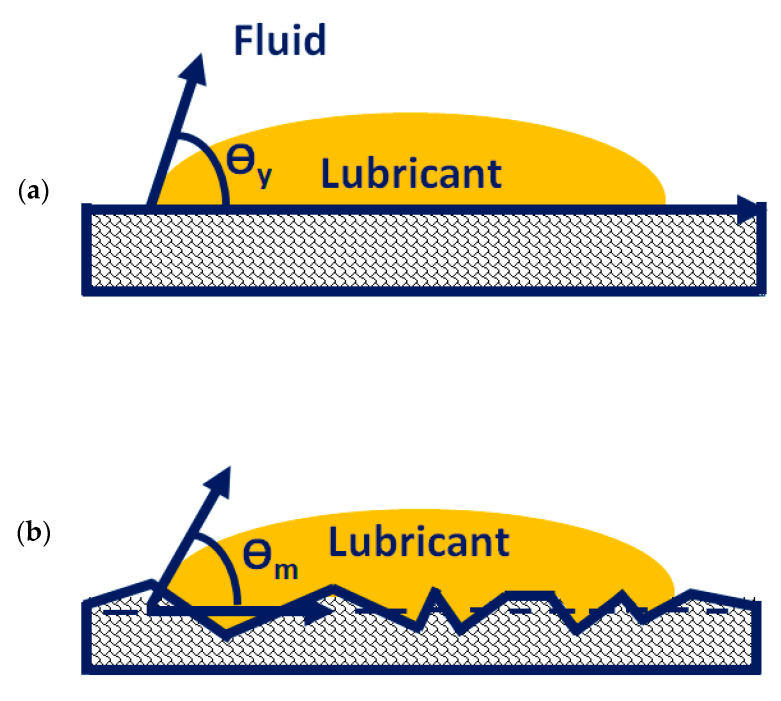
Definition of contact angle: (**a**) contact angle on ideal surface; (**b**) apparent contact angle on rough surface.

According to the Wenzel relation [42], increasing the surface roughness to a certain extent enhances the ability of the liquids to retain their contact with the surface (increase wettability). The WPC treatment process increases the surface roughness of the rollers by forming a fine micro-dimpled surface. This is one of the main reasons for the reduction in frictional losses. Due to the dimpled surface, an oil film is retained on the surface of the roller, which reduces the direct contact between cam and roller, and as a result, friction is reduced. The film’s retention is maximum at lower temperatures and weakens with the increase in temperature because at high temperatures the surface energy reduces, and as a result the bonding between the roller’s surface and the liquid film diminishes. As the surface roughness increases, the disruption of intermolecular bonds also increases, which leads to elevated surface energies. Because of the high surface energy, the surface tends to bond with the liquid molecules in order to minimize the energy, and liquids consequently achieve the complete wetting of surfaces. The increase in surface roughness also reduces the contact angle, i.e., the angle between the tangent to the liquid–vapor interface and the line that represents the apparent solid surface, Figure 17. This reduction in contact angle also increases surface wettability.

**Figure 18:**
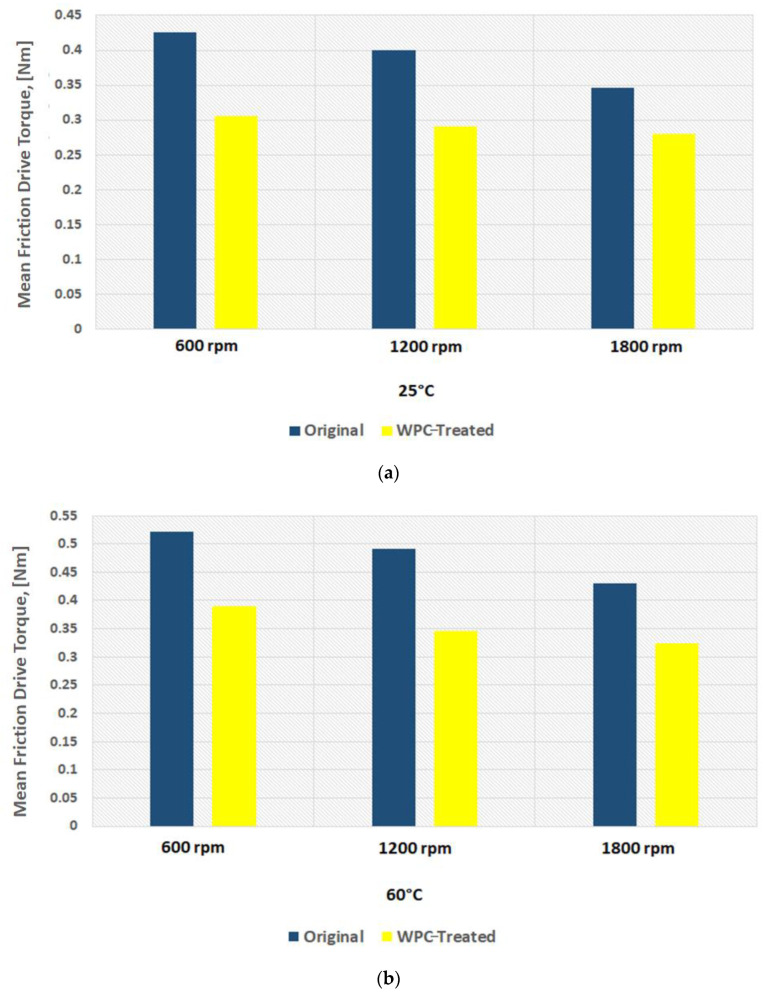

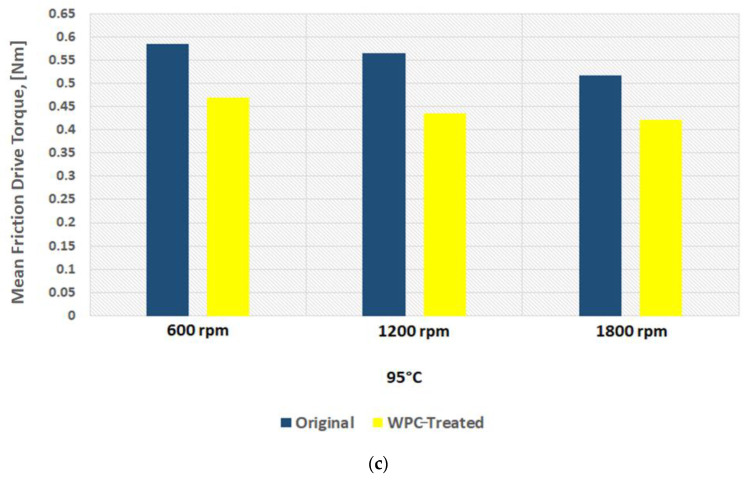
Mean friction torque at cam–roller interface at: (**a**) 25 °C; (**b**) 60 °C; (**c**) 95 °C.

The magnitude of measured frictional torque at the cam–roller interface under different camshaft speeds and oil inlet temperatures for both the original untreated roller and for the WPC-treated roller is displayed in Figure 18. The presented data is an average of ten complete engine cycles. It can also be observed clearly that the frictional losses of the WPC-treated roller are considerably less in comparison to the original untreated roller, as shown in Figure 18. At a lubricant inlet temperature of 25 °C and at cam speed of 600 rpm, the difference in frictional losses between the original and WPC-treated rollers is 28% (the friction torque is 0.43 Nm for the original roller and WPC-treated roller is 0.30 Nm). However, at a higher lubricant inlet temperature of 95 °C while operating on the same engine speed, the difference in frictional losses between the original and WPC-treated roller reduced by up to 19% (the friction torque is 0.59 Nm for the original untreated roller and for the WPC-treated roller it is 0.47 Nm). The reason for the reduction in friction for the WPC roller is attributed to the surface treatment, which relatively increased the micro-surface roughness of the roller by forming fine micro-sized dimpled surface, which helped retain the oil film on the roller’s surface. This retention of oil on the roller surface reduced the direct contact between the cam and roller, resulting in reduced friction.

Sliding at the roller–cam interface is principally regulated by the traction force of the interacting surfaces. The traction force is governed by multiple factors, which include the lubricant viscosity, lubricant temperature, lubricant formulation/chemistry, surface roughness of the mating parts, contact loading and pressure at the hydraulic lash adjuster [17,18,19,32,45,46,47]. Slippage of the roller normally occurs when the traction force is not able to keep the surfaces from rolling on each other [32]. It can be observed that an increase in rotational speed of the camshaft results in considerable reduction of frictional torque at the cam–roller junction as shown in Figure 18. It has also been previously reported that an increase in lubricant film thickness typically at lower operating temperatures at the roller–cam interface increases the tendency of roller slip because of weak traction force [47].

Lubricant viscosity decreases with the increase in temperature, resulting in a thinner lubricant film at higher operating temperatures and a thicker oil film at lower operating temperatures. At a higher temperature, the reduction in lubricant viscosity increases the tendency towards mixed and boundary lubrication at lower engine speeds, but as the rotational frequency increases, fluid film lubrication plays a greater role, and the lubrication regime shifts to one of hydrodynamic lubrication. Higher lubricant entraining velocities at any given temperature produce higher values of oil film thickness [17,18,19,32,46,47]. In the case of the original roller under a lubricant inlet temperature of 25 °C, the frictional losses reduced by 19% (from 0.43 Nm to 0.35 Nm) once the speed of the camshaft was increased from 600 rpm to 1800 rpm, as shown in Figure 18a. Low operating temperature and higher camshaft speed cause an increase in roller slippage under the influence of thicker lubricant film at the roller–cam contact zone. At a relatively higher operating temperature, the roller slip was reduced considerably due to the decrease in oil film thickness offering better traction force at the cam–roller interaction zone.

However, at a lubricant inlet temperature of 95 °C with the same increment in the speed, the frictional losses reduced by 12%, as shown in Figure 18c. A similar situation was also found for the WPC-treated roller, where the frictional losses were reduced by 8% and 10% at 25 °C and 95 °C, respectively, once the camshaft speed was increased from 600 rpm to 1800 rpm. The reason for this is that at higher frequencies, the lubrication conditions improve significantly at the cam–roller interface due to a decrease in contact load at the cam’s nose area [48] and a substantial increase in the quantity of lubricant entrained [17,18,19,32,46,47]. However, at reduced frequencies, the contact load at the cam’s nose is greatly affected by the compression of the valve spring, which causes higher friction around the cam’s nose region. This effect weakens at higher camshaft speed because of the inertial effects on the spring of the valve.

The effects of oil inlet temperature on the frictional torque at the cam–roller contact are realized in Figure 18. At a low camshaft operating speed of 600 rpm, the frictional torque increased by 27%—as shown in Figure 18a—once the temperature was increased from 25 °C to 95 °C, whereas the rise in friction reached up to 33% at a relatively high-speed of 1800 rpm as in Figure 18c. A similar trend of increase in frictional torque with rise in oil inlet temperature was also observed for the WPC-treated roller as shown in Figure 18. The reason for this is attributed to the fact that an increase in temperature reduces the oil’s viscosity considerably. The lubrication mode is pushed more towards the mixed and boundary regimes, increasing the tendency of asperity interactions, causing increase in friction at the cam–roller interface [17,18,19,32,46,47]. The experimental data shows a reduction in engine drive torque when using WPC-treated rollers in comparison to untreated/original rollers in any given testing condition. One of the main reasons for the reduction in drive torque of the WPC-treated rollers in comparison to the original untreated rollers is the increase in surface roughness by the WPC process; this increased average surface roughness has been known to help in lubricant film retention, which results in improved traction and also a reduction in roller slippage [32]. Traction is most probably improved by an increase in average surface roughness of the WPC-treated parts, which results in the prevention of slippage at the cam–roller interface and in turn reduces the drive torque. In addition to the phenomenon of increased lubricant retention obtained through the application of WPC-treatment, there are multiple other benefits of applying this process of mating metallic machine components and parts. The WPC process is protected by over 40 patents [34,39]; thus, limited information is available regarding the exact process. The process is known to provide multiple advantages, including strengthening of the metallic surface through thermal treatment and forging effects. Transformation into martensite of the austenite retained in the metallic surface layer is performed via WPC, resulting in a recrystallized, dense, high-toughness and high hardness surface [40], which not only reduces roller slippage [32] but also results in a reduction in drive frictional torque.

## 8. Conclusions

The engine head of a modern gasoline internal combustion engine was instrumented successfully for using an inline torque transducer to examine friction at the cam–roller interface under actual operating conditions. Little-to-no modification of components helped to capture the realistic friction data. The results revealed that frictional torque decreased up to 20% with the increase in camshaft operating speed from 600 rpm to 1800 rpm due to decreased contact load at cam’s nose region and enhanced lubrication conditions under the influence of higher entraining velocity. However, an increment of up to 33% in the value of valve train frictional torque was noted with an increase in oil inlet temperature. This is because of the reduction in lubricant viscosity resulting in a thin oil film at the cam–roller interface, thereby increasing the tendency of asperity interactions and a rise in friction at the cam–roller interface. Reduced frictional torque in the range of 8% to 28% was recorded for the WPC-treated roller in comparison to the original roller at different operating conditions, which indicated strong potential for the employment of surface treatment in the automotive sector/industry. The developed friction measurement technique and acquired experimental data can be very useful to further enhancing the tribological efficiency of engine valve trains in the future.

## Data Availability

Not applicable.
